# The influence of superstitions and emotions on villagers’ attitudes towards striped hyena in southwestern Iran

**DOI:** 10.1371/journal.pone.0285546

**Published:** 2023-08-08

**Authors:** Fatemeh Moures-Nouri, Mahmoud-Reza Hemami, Azita Rezvani, Benjamin Ghasemi

**Affiliations:** 1 Department of Natural Resources, Isfahan University of Technology, Isfahan, Iran; 2 Human Dimensions of Natural Resources Department & Center for Human-Carnivore Coexistence, Colorado State University, Fort Collins, CO, United States of America; U.S. Geological Survey, UNITED STATES

## Abstract

The intensity of human-carnivore conflict in socio-ecological systems may primarily be determined by people’s attitudes and perceptions of carnivore-related threats. Direct or indirect threats posed by large carnivores to human interests may eventually lead to negative attitudes that can trigger retaliatory bahaviour against them. We studied local people’s attitudes towards striped hyena (*Hyaena hyaena*), the nature and extent of the human-hyena conflict, and the socio-cultural drivers of the conflicts in 19 rural communities in southwestern Iran. We employed structural equation modelling to assess socio-cultural factors affecting attitudes towards striped hyenas. The findings of 300 interviews showed significant differences in local people’s superstitious attitudes regarding gender, age, and education. More than 40% of the participants had encountered hyenas, and on average, each respondent lost 0.44 livestock in the past five years due to hyena attacks. However, livestock depredation by the hyena was low (13.3%) compared to the damage inflicted by all carnivores (73%). While the respondents indicated some degrees of fear, hatred to hyena was relatively low and they generally showed positive attitudes towards the species. Women and older people expressed the highest and respondents with higher education the least superstitious beliefs. Attitude score of respondents toward hyenas was correlated negatively with hatred for hyenas and positively with knowledge about them, but socio-demographics effects on attitudes towards hyenas were not statistically significant. Self-reported livestock loss was a relatively good predictor of hatred and fear. Herders who had not protected their livestock reported carnivore attacks at least once. We conclude that superstitions can potentially negatively affect hyena persistence, but can be reduced by improving the educational level of local people.

## Introduction

Despite the ecological importance of carnivores, local communities’ negative attitudes towards these species often challenge conservation efforts [1]. Potential threats that carnivores impose on human lives and livelihoods (e.g. crop damage, livestock depredation and human injury/death) may lead to perceived risk and negative emotions [2], which can cause antagonistic behaviour against predators [3, 4]. As an essential mental capacity, emotion can be classified as cognition, conation, and affection [5] that influences human physiological state, expressions, and behaviour [6]. Large carnivores provoke strong conflicting emotions such as like, admiration, fear, and hatred [7]. Both positive and negative emotions toward carnivores may have important impacts on conservation and management programs [8]. Local people’s acceptance of conflict mitigation strategies about emotionally evocative carnivores depends on individuals’ emotions toward them [9, 10]. Consequently, the effectiveness and success of carnivore conservation efforts highly depend on cultural norms and beliefs that form such emotions and misconceptions towards species [11].

As scavenging carnivores, striped hyenas play a significant role in ecosystem functioning, providing important hygienic services by removing carcasses as sources of pathogens from the environment [12]. The striped hyena (*Hyaena hyaena*) distributed patchily in a vast geographic range from Africa to central Asia and India [13], but occurs at low population densities throughout its range, with many populations already extirpated [14, 15]. It is listed as Near Threatened on the IUCN Red List due to persecution, decreasing natural and domestic sources of carrion due to declines in the populations of other large carnivores including wolf (*Canis lupus*), cheetah (*Acinonyx jubatus*), leopard (*Panthera pardus*), lion (*Panthera leo*), and tiger (*Panthera tigris*), diminishing prey populations, and changes in livestock husbandry practices [14, 16–20]. The population of Iranian hyenas has also been decreasing as a result of poisoning, direct killing, and traffic accidents [21]. Striped hyenas mainly feed on carrion but may opportunistically predate on a variety of vertebrates and periodically supplement their diet with fruits and invertebrates [22]. Hence, they may cause damage to agricultural fields and livestock [23]. Understanding the impact of human activity on wildlife species is important to advocate human-wildlife coexistence. Dheer et al. (2022) have shown that under certain conditions anthropogenic activity may be compatible with the persistence of spotted hyena (*Crocuta crocuta*) [24]. Identifying the nature of conflict, local people’s attitudes toward wildlife, and the impact wildlife can impose on local communities’ livelihoods are equally important to managing human-wildlife conflict [25, 26]. People’s attitudes toward wildlife depend on their knowledge [27], emotions [28], beliefs [11], and experiences [29]. In addition, cultural aspects, including religion, traditions, myths, and superstitions, affect how people treat wildlife [30]. Therefore, considering social and cultural aspects affecting people’s attitudes toward wildlife species in human-wildlife conflict studies is important [31–33].

Additionally, perceived levels of conflict can differ from the actual levels due to the complex socio-psychological aspects of how people think and feel about wildlife [11] Locals may perceive carnivores as problematic species since they threaten their livelihood, regardless of their level of experienced conflict [34, 35]. Hence, people may feel more negative about a less harmful predator than those that have imposed damage [11].

Anthropogenic threats have been identified as a major cause of striped hyena mortality [16]. Historically, hyenas, especially striped hyenas, have elicited numerous superstitious beliefs in different cultures worldwide [36]. Hyena body parts have been widely used and exploited to cure diseases by humans [16, 37]. Like an animal to which magic is attributed, the hyena has greatly aroused people’s imagination in Africa and Asia. In West and South Asia, hyena’s organs are used as magic, spells, charms, or talismans [38]. For instance, dried striped hyena skin has been used as a powerful spell in Iran [39]. In Afghanistan and Pakistan, striped hyena hair is used to treat disease and for the magic of love [40]. Hyena blood has been exploited as a potent medicine in northern India, and eating hyena tongue has been believed to help fight tumors [38]. Palestinian people associated hyenas with supernatural forces [41], and in Jordan, it is believed that hyenas love human food and kill their prey to eat at their leisure. People in the Arabian Peninsula and Northern Africa have believed that the striped hyena’s right ear has healing properties against many diseases, but its left ear is toxic [16]. From the local people’s perspective in Khojir National Park in Iran, the striped hyena is a powerful, scary, impure, and cannibalistic animal that could assault or abduct children [42]. In ancient Greece and Rome, blood, excrements, rectum, genitalia, eyes, tongue, hair, skin, and fat, as well as the ash of different parts of the body were used as a means to repelling evil, ensuring love and fertility [43–45]. Because of these beliefs, indiscriminate killings of striped hyenas have happened throughout its range [42, 46, 47], which is a credible threat to its persistence.

In this study, we aimed to understand the nature and extent of the human-hyena conflict and determine socio-cultural factors affecting attitudes towards striped hyenas in rural communities in Dezful County, southwestern Iran. As the study’s theoretical framework we hypothesised that 1- the amount of self-reported livestock loss to hyenas, negative emotions for them, and superstitious beliefs about them affect respondents’ attitudes towards hyenas, 2- individuals’ knowledge about the species would positively affect attitudes towards hyenas.We controlled for the effects of demographics on all our model variables.

## Materials and methods

### Study area and survey design

**Fig 1:**
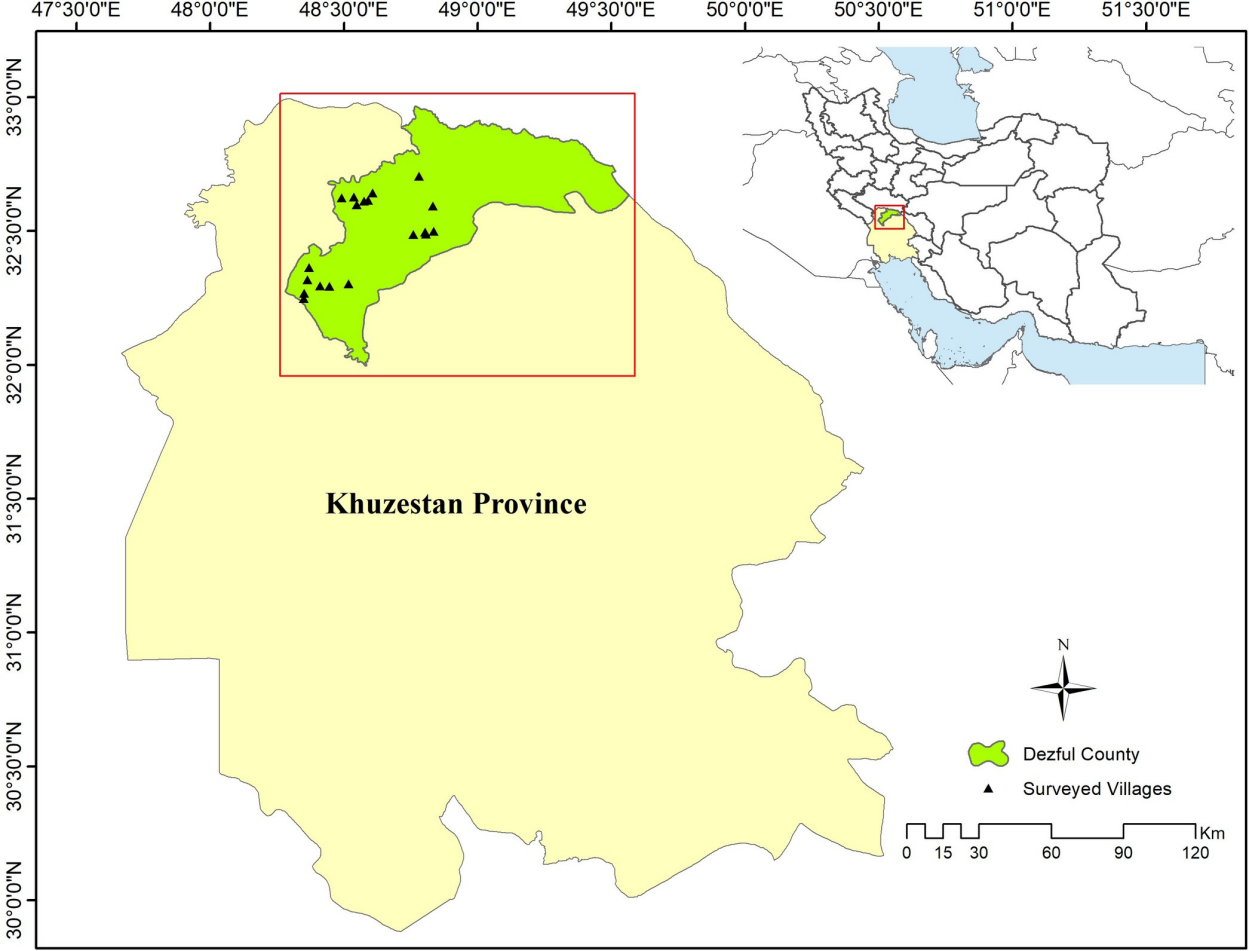
The position of the study area (Dezful county) on Iran’s map with locations of the 19 villages surveyed.

We surveyed rural areas in the central and southwestern parts of Dezful county in Southwestern Iran (Fig 1). The study area was selected based on the high number of human-hyena conflicts reported to the Department of Environment. Three hundred participants were interviewed in 19 villages from November 2018 to February 2019. The villagers were mainly pastoralist herders and farmers. We randomly selected the participants in each village and later adopted snowball sampling. The study including its ethical considerations was approved by the educational office of the Department of Natural Resources, Isfahan University of Technology, Iran (letter number code 121.98.18678 dated 21 July, 2019). We also got permission from the Dezful Department of Environment for performing the survey. Informed verbal consent obtained from the participants after giving explanations about the purpose of the research, the topic of different parts of the questionnaire, and how the interview will proceed. The interviews proceeded in a non-formal and trustful atmosphere in Persian (the mother language of the participants) and data were collected anonymously. Only individuals aware of hyena’s existence in the area were interviewed.

Our questionnaire comprised five sections to examine (1) socio-economic status; (2) the severity of hyena’s damage to crops, livestock, and poultry; (3) respondents’ knowledge about hyenas; (4) superstitious beliefs and feelings about hyenas; and (5) attitudes toward the species (S1 Text). To evaluate the face validity of the questionnaire, we performed 30 pre-test interviews with locals. According to the collected information, the questionnaire was slightly modified, and a few questions were removed.

We asked respondents about their gender, age, education, livestock ownership, and livestock loss to hyenas. Questions regarding knowledge about hyena consisted of its diet, life history, and ecology and were provided with response scales from "strongly disagree" (1) to "strongly agree" (5). In addition, we elicited respondents’ superstitious beliefs about hyenas using eight items developed for this study based on expert opinions. We also measured positive attitudes towards hyenas using six items adapted from [48]. Responses were anchored using response scales from "strongly disagree" (1) to "strongly agree" (5). Finally, we measured respondents’ feelings of hatred and fear for hyenas providing four response categories from "nothing" (0) to "high" (3).

Knowledge scores for respondents were calculated as the average score for the eight items assessing knowledge about hyenas on a response scale from "strongly disagree" (1) to "strongly agree" (5). Last, we operationalised self-reported loss to hyenas as the ratio of the reported loss in the past five years to the total number of sheep and goats owned by the respondent.

### Analysis

We evaluated the internal consistency of our latent constructs using Cronbach’s alpha coefficient [49]. To analyse the hypothesised model, we followed a two-step procedure for structural equation modeling SEM, [50]. First, we tested the measurement model using confirmatory factor analysis (CFA). After establishing a satisfactory measurement model, we proceeded to test the structural relationships.

All SEM-related analyses were conducted using Mplus 8.3 [51]. We used the full information maximum likelihood (FIML) method of estimation to handle missing data and robust maximum likelihood estimation (MLR) to deal with data non-normality in SEM [52]. Model fit adequacy was assessed according to [53] recommendations: Root Mean Squared Error of Approximation (RMSEA) values close to 0.06, Tucker-Lewis Index (TLI) and Comparative Fit Index (CFI) values close to 0.95, and Standardised Root Mean Squared Residual (SRMR) values close to 0.08. All non-SEM analyses were conducted in SPSS 25 [54]. Statistical significance was evaluated at minimum 0.05 level.

## Results

### Sample characteristics

The average age of respondents was 51.6 years old (SD = 10.8), and 46 out of 300 were female (15.3%). One respondent had a bachelors’ degree, 39 high school diplomas, 150 elementary schools diplomas, and 110 were illiterate. We interviewed 233 herders and farmers (77.7%); of these, 63 (27%) were herders, 145 (62.3%) were farmers, and 25 (10.7%) were both farmers and herders. The rest of the interviewees (67 people) had other occupations. A total of 95 interviewees owned livestock. Mean number of livestock per respondent including those who were not herders ± SD was as follow: sheep (*Ovis aries*): 13.16 ± 2.12; goat (*Capra aegagrus hircus*): 8.75 ± 1.51; cow (*Bos taurus*): 0.63 + 3.21; buffalo (*Bubalus bubalis*): 0.06 + 0.41; horse (*Equus caballus*): 0.02 + 0.22); donkey (*Equus asinus*), 0.01 + 0.17). The minimum number of livestock among the people who owned livestock was 25, and the maximum was 140.

### Perceived conflicts and attitudes

Respondents reported conflict with all the existing large carnivores including wolf, leopard, and jackal (*Canis aureus*) in the study area. However, 82 people (27%) did not report any damage from the carnivores. Of those having conflict with carnivores, only 29 people (13.3%) reported damage from hyena. Thirty-one interviewees (49.2%) whose sheepfold had a metal or solid fence did not report any hyena attack. In contrast, 32 livestock owners (50.8%) lacked a fence around their sheepfolds. To protect their livestock against carnivores, 14 people of the latter group used to use dogs (43%), traps (21.4%), weapons (21.4%), guards (7.1%), and poison (7.1%); while, the rest (n = 18) did not use any of these control methods. Of those who experienced carnivores’ damage (n = 218), only 12 people (5.5%) submitted a complaint to the Department of Environment.

**Fig 2:**
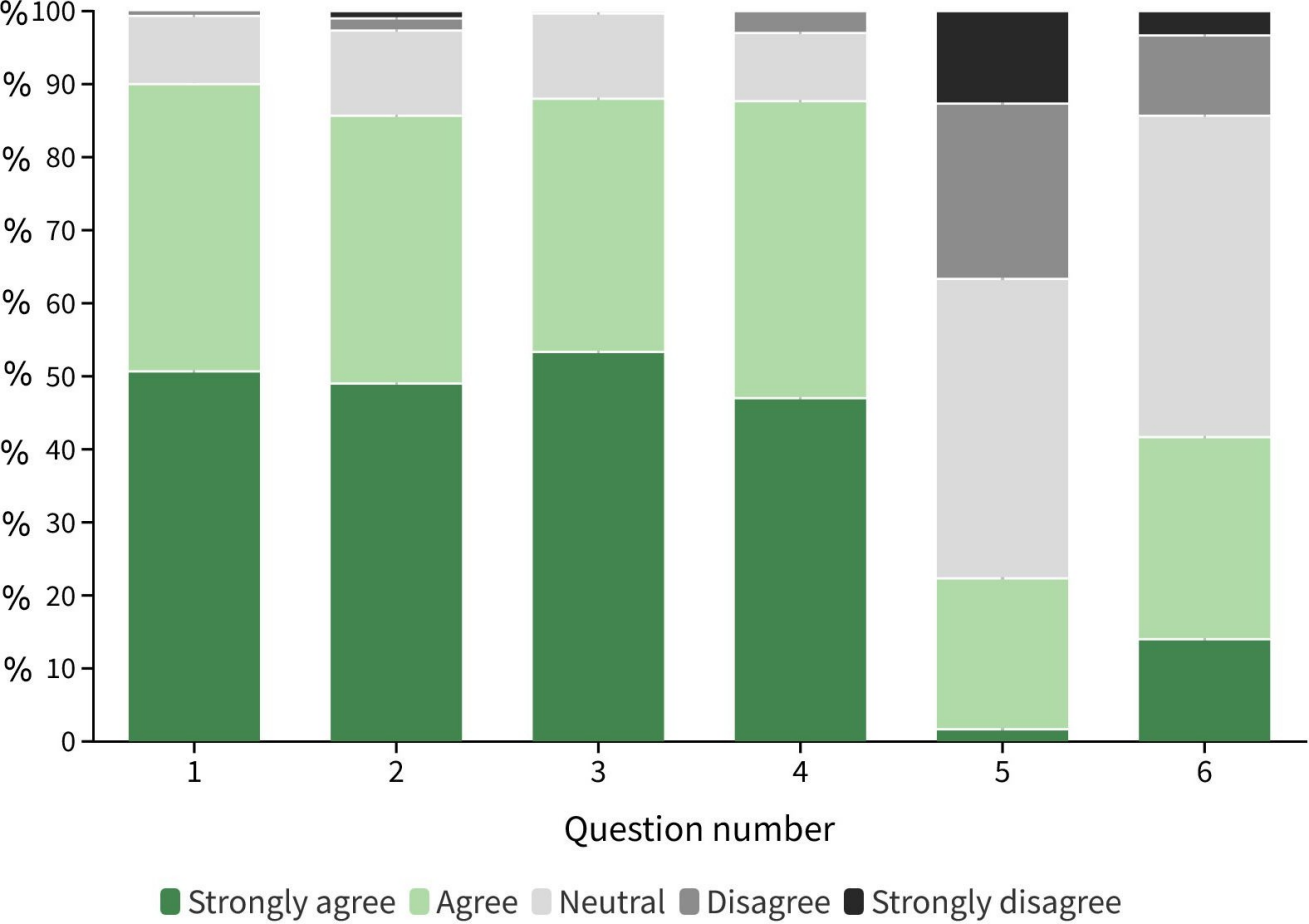
Stacked bar chart representing frequency distribution (in percentages) of different answers to the questions measuring local people’s attitudes toward striped hyenas, as shown in the first section of Table 1.

Descriptive statistics of responses to each item used in the analysis are provided in Table 1. Respondents, on average, agreed with statements indicating their positive attitudes towards hyenas (Fig 2). On average, hatred for hyenas was small (Median = low), while respondents indicated a moderate amount of fear for hyenas (Median = neutral). Finally, on average, each respondent reported 0.44 sheep or goats lost to hyenas (SD = 1.34) over the past five years combined.

**Table 1:** Item wordings and descriptive statistics, factor loadings, and scale reliability values.

Construct	Median	Factor Loading ^*b*^	Cronbach’s Alpha
***Attitudes*** ^***a***^		*n*.*a*.	
1. Hyenas, like other species, have the right to live in their natural habitat.	strongly agree	.95	0.86
2. I like to teach my children about hyenas at school.	agree	.84	
3. I would like the hyena to stay in the nature of Dezful.	strongly agree	.98	
4. By eliminating hyenas, we can prevent damage to livestock/farm (reverse coded).	agree	.50	
5. Hyenas must be removed by the DoE (reverse coded).	neutral	.43	
6. The DoE must allow people to fight hyenas (reverse coded).	neutral	.47	
*Hatred*	low	n.a.	n.a.
*Fear*	neutral	n.a.	n.a.
***Superstitions*** ^***a***^		*n*.*a*.	
1. Some parts of the hyena’s body are used to treat diseases.	neutral	.97	0.96
2. Some parts of the hyena’s body bring good luck.	neutral	.96	
3. Some organs of the hyena increase sustenance.	agree	.91	
4. Killing a hyena increase a blessing for the people of the region	neutral	.90	
5. The hyena is one of the most aggressive and cannibalistic predators	disagree	.52	
6. The use of female hyenas’ genitals is effective in treating infertility	neutral	.89	
7. Hyena hair reduces headache	neutral	.80	
8. The effect of prayer is greater when it is written on the skin of a hyena	neutral	.78	
***Knowledge***			
1. Hyenas do not build nests and use natural holes and caves as nests	agree	n.a.	n.a.
2. Hyenas are more active at night	strongly agree		
3. Hyenas escape and hide when they encounter humans	neutral		
4. Hyenas collect bones and some objects	agree		
5. Hyenas are seen in groups of less than four	agree		
6. Hyena is a useful animal and does not harm humans	neutral		
7. Hyenas clean the environment	neutral		
8. Hyenas can be seen in most parts of Iran	agree		
***Self-reported loss***		n.a.	n.a.

^a^Latent variable means and standard deviations reflect the grand means of the corresponding items.

^b^Standardised factor loadings, all significant at .001 level.

### Structural equation modelling

Based on Cronbach’s alpha coefficients, our two latent constructs (i.e., attitudes towards hyenas and superstitious beliefs about them) had good internal consistency (α > 0.7 Table 1); [49]. Our measurement model consisted of all the latent and observed variables. Based on modification indices, we added seven covariances between indicators, all within the same latent construct, to improve the measurement model fit. The final measurement model showed acceptable fit to the data [χ2 (df) = 429.62 (153), CFI = 0.935, TLI = 0.911, RMSEA = 0.078, SRMR = 0.076]. All factor loadings were above the 0.4 threshold and were statistically significant at the 0.001 level (Table 1) [55].

**Fig 3:**
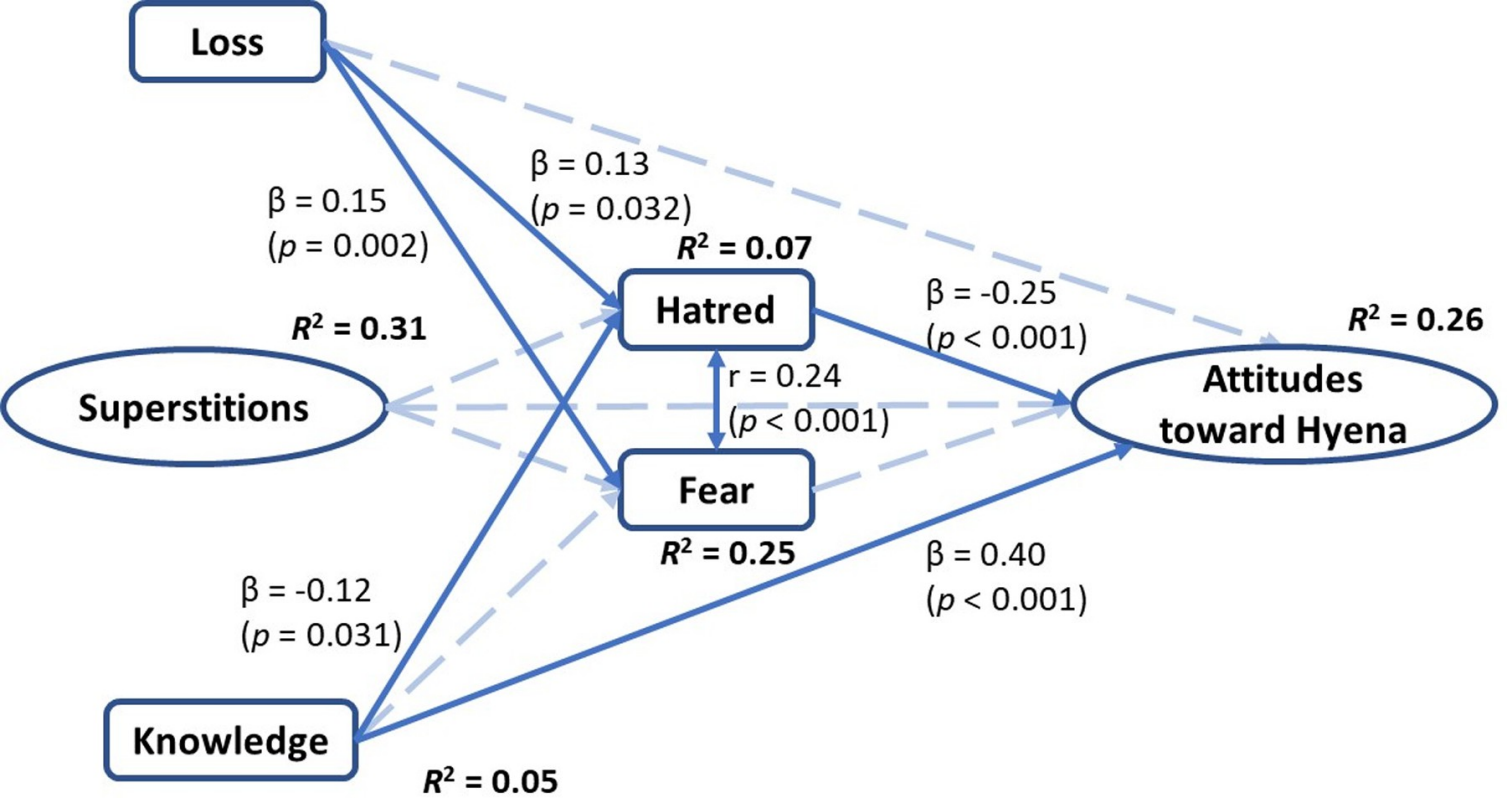
Results of the latent variable structural equation modelling. Path labels are standardised coefficients. Single-arrow lines represent regression paths, and double-arrow lines indicate a reciprocal correlation.

After establishing our measurement model’s adequacy, we tested the hypothesised structural relationships. Our structural model showed acceptable fit to the data [χ2 (df) = 432.75 (156), CFI = 0.930, TLI = 0.908, RMSEA = 0.077, SRMR = 0.077]. Fig 3 presents a graphical summary of the standardised regression coefficients for the three models, and the effects of control variables (i.e., age, education, and gender) on each endogenous variable in the model are presented in Table 2.

**Table 2:** Effects of demographic variables on each variable in the model.

			Endogenous variable		
Exogenous variable (control)	Attitudes	Hatred	Fear	Superstitions	Knowledge
Age	0.10 (*p* = 0.273)	-0.20 (*p* = 0.037)	-0.36 (*p* < 0.001)	0.24 (*p* = 0.005)	0.23 (*p* = 0.031)
Education	0.10 (*p* = 0.263)	-0.06 (*p* = 0.580)	0.06 (*p* = 0.480)	-0.24 (*p* = 0.005)	0.27 (*p* = 0.010)
Gender (female)	-0.02 (*p* = 0.774)	-0.13 (*p* = 0.031)	0.34 (*p* < 0.001)	0.24 (*p* < 0.005)	-0.10 (*p* = 0.087)

The hypothesised model explained 26% of the variance in attitudes towards hyenas. Hatred for hyenas was negatively related with attitudes (β = -0.25, *p* < 0.001), while knowledge about hyenas positively predicted attitudes towards them (β = 0.40, *p* < 0.001). Self-reported loss to hyenas statistically significantly predicted hatred (β = 0.13, *p* = 0.032) and fear (β = 0.15, *p* = 0.002) for them. Moreover, knowledge about hyenas was negatively associated with hatred for them (β = -0.12, *p* = 0.031). Last, hatred and fear for hyenas were statistically significantly correlated (*r* = 0.24, *p* < 0.001). The relationships between superstitions and hatred (γ = 0.05, *p* = 0.470), fear (γ = 0.08, *p* = 0.191), and attitudes (γ = 0.06, *p* = 0.357), as well as the relationships between loss and attitudes (γ = -0.05, *p* = 0.376) and knowledge and fear (γ = 0.03, *p* = 0.541) were not statistically significant.

Older respondents expressed less fear (γ = -0.36, p < 0.001) and hatred (γ = -0.20, *p* = 0.037) for hyenas, while their knowledge (γ = 0.23, *p* = 0.031) and superstitious beliefs about hyenas (γ = 0.24, *p* = 0.005) were higher than younger individuals. Respondents with more education held less superstitious beliefs about hyenas (γ = -0.24, *p* = 0.005) and more knowledge about them (γ = 0.27, *p* = 0.010). Female respondents showed more fear (γ = 0.34, *p* < 0.001) and less hatred (γ = -0.13, *p* = 0.031) for hyenas than males, and held more superstitious beliefs (γ = 0.24, *p* < 0.001). Finally, while we had controlled all the predictor variables for age, education, and gender, the relationship between socio-demographic variables and attitudes towards hyenas were not statistically significant (Table 2).

## Discussion

Striped hyenas in many rural areas across Iran and elsewhere are threatened by people’s superstitious beliefs, where they are persecuted for damage prevention and obtaining folk remedies [16, 36–42]. We examined the effect of socio-cultural factors on locals’ attitudes toward this species. Our hypothesised structural model investigated the effect of reported loss to hyenas, knowledge of the species, and negative emotions (i.e., fear and hatred) on attitudes towards hyenas while controlling for demographic variables. This study is among the first ones that utilised quantified data on human-hyena conflict in Iran for improving future conservation strategies.

In accordance with the results in the literature, e.g. Mordi [56] and Drews [28], respondents’ knowledge of hyenas positively predicted attitudes towards them. Moreover, respondents with higher knowledge of hyenas expressed less hatred for the species, which led to a decrease in their negative attitudes towards hyenas. These results suggest that awareness-raising and educational efforts can promote the conservation of hyenas among local communities, which is in line with previous studies [57].

Reported loss to hyenas was not directly related to attitudes towards the species. However, loss to hyenas contributed to the hatred and fear for them, and hatred had a negative effect on attitudes. This finding is supported by the literature that suggests actual loss to carnivores has little direct effect on attitudes compared to social and psychological factors [28, 29].

In contrast to our expectations, the hypothesised relationships between superstitions and negative feelings toward hyenas and attitudes towards them were not statistically significant. We speculate this might be since the superstitious beliefs we asked about in the study had mixed valences, some of them being neutral, negative, or positive. For instance, while we asked about respondents’ superstitions about negative traits of hyenas (e.g., cannibalism and aggression), other items were about remedial effects of hyena body parts that do not necessarily imply negative connotations. However, as our results revealed a considerable proportion of the local people believed in medicinal value of hyena’s organs. Hence, there may still be a demand for the hyenas’ ongans in local communities, which can threaten the survival of the species.

Concerning demographic variables, more educated respondents held less superstitious beliefs and more knowledge of hyenas. This is in accord with the literature that suggests macro-level socio-economic factors can influence attitudes towards carnivores [11, 58, 59]. With global trends toward modernisation and progress in human development, future generations will likely hold more positive attitudes toward the conservation of carnivores [60, 61]. Moreover, older respondents expressed less negative emotions and more superstitious beliefs and knowledge of hyenas. The greater prevalence of superstitions among older people could be due to higher illiteracy rate among them. This emphasises the importance of education for preventing transmission of superstitions to the next generation.

### Perceived conflicts

Livestock is considered an important source of income and savings and plays a significant role in local people’s economic status. Therefore, carnivore attacks on livestock are a major problem for local peoples. In our study, locals reported hyena’s damage to livestock, but not crops. However, damage to both livestock and crops is documented in other studies [16, 62]. Consistent with Osborn and Helmy [63] findings, hyenas’ economic damage seems small, and livestock can be protected by proper fencing.

A limited number of local people took advantage of fencing, poisons, weapons, traps, dogs, and guards to protect their livestock against predators. On the other hand, many people did not employ any of these controlling methods. The interview findings showed that those who had fenced sheepfolds did not report carnivore attacks. Unlike felids and wolves, which are capable of penetrating most roofless sheepfolds [64], fencing sheepfolds can easily keep hyenas from entering and damaging livestock.

### Attitudes

In accordance with previous studies [65–67], we found that local people’s superstitious attitudes toward hyenas are affected by variables such as gender, age, and education [65, 66, 67]. The superstitious attitude of older people was higher than younger ones, and again low education level of older people may have been responsible for shaping such beliefs. Higher education levels result in a less negative attitude towards conservation actions [68]. In our study area, some local people, especially farmers, believed that the beheading hyena was lucky. If they bury the hyena’s head on their farm, it will increase their livelihood and have a productive year. A number of locals also believed that body parts of this species are accompanied by luckiness and are useful in treating diseases. On the contrary, some people mentioned that this animal brings bad luck and ominousness to the region. Similar superstitious attitudes toward hyenas were reported from villages near Khojir National Park in the vicinity of Tehran, Iran [42].

Age also played a remarkable role in local people’s conservation perspectives, with young people having a relatively more positive attitude, in line with the findings of [60, 61]. Most people that suffered damage from hyenas showed a negative attitude toward this animal. People have rarely been present at the moment of hyena attacks. Hence, some of the reported attacks may have been done by other carnivore species. About half of the interviewed people were not confronted by hyenas, but they hated them. Considering that most retaliatory activities done by people are resulted from their feelings, such as fear and hate, declining their hatred may decrease hyenas’ killing.

### Conservation implications

Education and public awareness programs could increase knowledge and subsequently correct diagnosis of the problem species [69] and transform local attitudes toward wildlife conservation [60, 70, 71]. Lack of awareness about conservation matters and exclusion of people from decision-making may lead to negative attitudes [72, 73]. Therefore, considering local people’s attitudes could assist in making proper managerial decisions and resolving conflict. Only a small portion of the locals who have suffered damage from wildlife reported the cases to the Department of Environment. The reason behind locals’ unwillingness to report wildlife attacks may be the non-payment of damages by DoE or non-serious damage. Compensation for damage could enhance the conservation attitude of people [74]; however, the efficiency of compensation in controlling conflicts must be validated [75, 76]. Our results revealed that fenced sheepfolds could effectively prevent hyenas’ penetration; hence, barrier fencing to separate hyenas from livestock could lessen damage and conflict.

## Supporting information

S1 Text Questionnaire for the survey of attitudes towards striped hyena in Dezful county. (We used Farsi version of the questionnaire in interviews). (DOCX)

S1 File (TXT)

S1 Data (XLSX)
