# Supplementary material for: The influence of superstitions and emotions on villagers’ attitudes towards striped hyena in southwestern Iran
Source: PLoS One. 2023 Aug 8;18(8):e0285546. doi: 10.1371/journal.pone.0285546 (PMC10409376; doi:10.1371/journal.pone.0285546)
Supplement: S1 Text — (DOCX) [file pone.0285546.s001.docx]

S1 text. Questionnaire for the survey of attitudes towards striped hyena in Dezful County. (We used Farsi version of the questionnaire in interviews)

***Locals' socio-economic background***

Age: _____

Sex: male female

Education: _____

Source of livelihood: _____

***Conflicts with carnivores***

Have you ever encountered hyenas? Yes No

Do the hyenas have damaged your animals/crops? Yes No

| Livestock type | Number of livestock | Number of predated livestock by hyena in the last 5 years |
| --- | --- | --- |
| Sheep |  |  |
| Goat |  |  |
| Cow |  |  |
| Buffalo |  |  |
| Donkey |  |  |
| Horse |  |  |

In what season do most hyena damages happen?

What other large carnivores are you in conflict with?

What problems have each of them caused?

What do you do to avoid damages by hyenas?

Has a hyena ever managed to break into your animal pen?

What material is that animal pen made of?

Have you reported any problem with the hyena to the DoE?

If no, why not?

***Knowledge of local people towards hyenas***

|  | strongly disagree | disagree | neutral | agree | strongly agree |
| --- | --- | --- | --- | --- | --- |
| 1-Hyenas use natural holes and caves as nests. |  |  |  |  |  |
| 2-Hyenas are more active at night. |  |  |  |  |  |
| 3-Hyenas escape and hide when they encounter humans. |  |  |  |  |  |
| 4-Hyenas collect bones and some objects. |  |  |  |  |  |
| 5-Hyenas are usually seen solitary or in groups of less than four. |  |  |  |  |  |
| 6- Hyenas are useful to human beings. |  |  |  |  |  |
| 7- Hyenas clean the environment. |  |  |  |  |  |
| 8- Hyenas can be seen in most parts of Iran. |  |  |  |  |  |

***Attitudes of local people towards hyenas:***

| ***Superstitious attitudes*** | | | | | |
| --- | --- | --- | --- | --- | --- |
|  | strongly disagree | disagree | neutral | agree | strongly agree |
| 1- Some parts of the hyena’s body are used to treat diseases. |  |  |  |  |  |
| 2- Some parts of the hyena's body bring good luck |  |  |  |  |  |
| 3- Some organs of the hyena increase sustenance. |  |  |  |  |  |
| 4- The hyena is one of the most aggressive and cannibalistic predators. |  |  |  |  |  |
| 5- The use of female hyenas (genitals) is effective in treating infertility. |  |  |  |  |  |
| 6- Hyena hair reduces headache. |  |  |  |  |  |
| 7- The effect of prayer is greater when it is written on the skin of a hyena. |  |  |  |  |  |

| ***Fear and hatred*** | | | | |
| --- | --- | --- | --- | --- |
|  | Nothing | low | neutral | high |
| How do you express your feelings (fear) toward hyenas? |  |  |  |  |
| How do you express your feelings (hatred) toward hyenas? |  |  |  |  |

| ***Conservation attitude*** | | | | | |
| --- | --- | --- | --- | --- | --- |
|  | strongly disagree | disagree | neutral | agree | strongly agree |
| 1. Hyenas, like other species, have the right to live in their natural habitat. |  |  |  |  |  |
| 2. I like to teach my children about hyenas at school. |  |  |  |  |  |
| 3. I would like the hyena to stay in the nature of Dezful. |  |  |  |  |  |
| 4. By eliminating hyenas we can prevent damage to livestock/farm. |  |  |  |  |  |
| 5. Hyenas must be removed by the DoE. |  |  |  |  |  |
| 6. The DoE must allow people to fight hyenas. |  |  |  |  |  |
